# Assessment of the Reach, Usability, and Perceived Impact of “*Talking Is Power*”: A Parental Sexual Health Text-Messaging Service and Web-Based Resource to Empower Sensitive Conversations with American Indian and Alaska Native Teens

**DOI:** 10.3390/ijerph18179126

**Published:** 2021-08-30

**Authors:** Lea Sacca, Stephanie Craig Rushing, Christine Markham, Ross Shegog, Melissa Peskin, Belinda Hernandez, Amanda Gaston, Michelle Singer, Nicole Trevino, Chrystial C. Correa, Cornelia Jessen, Jennifer Williamson, Jerri Thomas

**Affiliations:** 1 Center for Health Promotion and Prevention Research, The University of Texas Health Science Center at Houston, Houston, TX 77030, USA; christine.markham@uth.tmc.edu (C.M.); ross.shegog@uth.tmc.edu (R.S.); melissa.f.peskin@uth.tmc.edu (M.P.); chrystial.c.correa@uth.tmc.edu (C.C.C.); 2 Northwest Portland Area Indian Health Board, Portland, OR 97201, USA; agaston-contractor@npaihb.org (A.G.); msinger@npaihb.org (M.S.); info@nicoletrevino.com (N.T.); 3 School of Public Health, The University of Texas Health Science Center at San Antonio, San Antonio, TX 78229, USA; belinda.hernandez@uth.tmc.edu; 4 Alaska Native Tribal Health Consortium, Anchorage, AK 99508, USA; cmjessen@anthc.org (C.J.); jjwilliamson@anthc.org (J.W.); 5 Inter Tribal Council of Arizona, Inc., Phoenix, AZ 85004, USA; jerri.thomas@itcaonline.com

**Keywords:** AI/AN youth, AI/AN parents, sexual health, parent-child communication, text message service, informed decision-making

## Abstract

Background: Early sexual debut among American Indian and Alaska Native (AI/AN) adolescents has been associated with an increased risk of teenage pregnancies and sexually transmitted infections, along with an increased risk of having multiple lifetime sexual partners, and engaging in greater frequency of sex, substance abuse, and lack of condom use. A major protective factor against early sexual debut among AI/AN youth is the familial system. Interventions aiming to improve parent–child communication and parental warmth toward adolescent sexual health topics were reported to contribute to positive youth sexual health outcomes, specifically among minority youth. Healthy Native Youth thus developed the Talking is Power text-messaging service to guide parents and caring adults on how to initiate sensitive topics with youth and how to support them in making informed decisions regarding sex and healthy relationships. Methods: Descriptive statistics were used to demonstrate website analytics and reach per views and time spent on each page, and for displaying participants’ responses to the questions on the usability of the Talking is Power text-messaging series. To assess the perceived impact of the series, the differences in mean percentage scores of the question assessing parental comfort in engaging in sexual health topics with youth between pre- and post-intervention were calculated using two-sample *t*-tests of equal variances. Descriptive content analysis was adopted to highlight emerging themes from open-ended items. Results: When looking at reach, 862 entrances were recorded during the specified time period (5.8% of total entrances to HNY website), while the bounce rate was set at 73.1% (22.6% greater than the industry average), and the exit rate was 54.3% (15.2% greater than the industry average). Series usability was highly ranked on the 5-Likert scale in terms of signing up for a similar series on a different topic, quality of images, texts, and links, relating to prompts, and change in sparking sensitive conversations with youth. High likelihood of recommending the series to a friend or colleague was also reported by participants (0–10). No significant difference in parental comfort levels was reported (*p* = 0.78 > 0.05). Main themes provided suggestions for improving the series mode of delivery, while others included positive feedback about the material, with the possibility of expanding the series to other adolescent health topics. Conclusion: Lessons learned during the design, dissemination, and evaluation of the resource’s usability, reach, and perceived impact may be of interest to other Indigenous communities who are in the process of adapting and/or implementing similar approaches.

## 1. Background

Systemic inequities, including the lack of sufficient and culturally appropriate sexual health education, have placed American Indian and Alaska Native (AI/AN) adolescents at higher likelihood of engaging in risky sexual behaviors that, in turn, result in adverse sexual and reproductive health outcomes [1]. In 2017, AI/AN females 15 to 19 years old ranked first nationwide in teenage pregnancies [2]. The birth rate among AI/AN youth was reported at 32.9 per 1000 individuals compared to 13.2 among White adolescents [2]. AI/AN youth 15 to 19 years old also had the highest prevalence of repeat teen birth (21.6%) in 2010 compared to 20.9%, 20.4%, and 14.8% among their Hispanic, Black, and White counterparts, respectively [3]. Native young adults and adolescents also suffer from disproportionately higher rates of chlamydia and gonorrhea compared to other racial/ethnic groups [2].

Various socioecological factors influence the sexual and reproductive health of AI/AN youth, including residing in a remote rural area, experiencing poverty, dealing with substance misuse, lacking insufficient access to sexual health education, having poor access to reproductive health services in local clinics, and social stigmas related to sex, sexual violence, and historical trauma [4]. Native communities may also have favorable views toward the arrival of new life regardless of the parent’s age. Such traditional and contemporary cultural values influence and shape AI/AN youth’s sexual and reproductive health decisions [5]. To meet youth where they are, sexual health messaging must reflect the cultural values, social contexts, and health epistemologies of tribal communities to produce desired health outcomes [6].

Adolescence is usually identified as a developmental period related to some degree of psychosocial vulnerability [7]. Particularly, parents may be worried by some difficulties of their adolescent children related to problems such as substance use [8] or problems in psychosocial adjustment [9]. Adolescence may be viewed differently in Western cultural contexts [10,11] compared to Eastern cultural contexts [12], as well in ethnic minority contexts [13]. Parental monitoring and parent–child relationship quality serve as buffers against negative health adolescent health outcomes only when adolescents perceive a strong parent–child relationship, which allows them to avoid negative consequences associated with substance use and similar adverse behaviors [14]. In Native communities, protective factors against substance use include individual characteristics and experiences, as well as social contexts that are likely to inform preventive efforts [15].

Similar to substance use, a major protective factor against early sexual debut among AI/AN youth is the familial system [16]. Nationally, across racial/ethnic groups, more than 50% of teens aged 12–15 report their parents as their main influence when making decisions about sex. Teens aged 16–19 also consider their parents as their main influence when making these kinds of decisions [17]. Greater perceptions of parental monitoring and parent–child communication about sexual health topics were identified as key factors in delaying the early initiation of sex among AI/AN youth [18,19]. These factors were also highlighted as effective strategies in sexual health promotion interventions involving both parents and children [20,21]. For instance, a systematic review on parent-based adolescent sexual health interventions carried out by Santa Maria et al. (2015) highlighted improvements in sexual health communication among minority parents participating either in group sessions or through self-paced activities. Out of eleven controlled trials analyzed for effectiveness, a significant impact was seen on increasing communication (Cohen’s d, 0.5), while the analysis of nine trials showed a large effect on increasing parental comfort with communicating about sexual health topics (Cohen’s d, 0.7). Intervention participants experienced positive communication outcomes regardless of delivery mode or intervention dose. Compared to controls, participants were 68% and 75% more likely to report improved communication and increased comfort, respectively [22].

The use of media technologies and social platforms by AI/AN adults has improving greatly over the past decade [23]. To bridge the digital divide affecting Internet access, the Indian Health Service (IHS) has focused on developing health information technology and telemedicine, as well as on culturally tailored prevention and disease management programs to enhance health equity [23]. Electronic health records and telehealth services are two major tools that are now being used to improve the delivery of comprehensive health services for AI/AN adults, using community-centered initiatives [24].

High engagement in online evidence-based interventions has been reported among AI/AN adults [24,25,26]. A web-based therapeutic intervention developed to reduce PTSD symptoms through psychoeducation, symptom self-management tools, and community-based participatory research activities showed that the majority of AI/AN adult participants (86%) perceived the culturally adapted website and associated text message reminders as satisfactory and effective [25]. Moreover, 55% of participants reported using the website at the recommended intensity at six weeks from the program initiation [25]. A qualitative analysis of the perspectives of AI/AN stakeholders with type 2 diabetes (T2D) receiving online diabetes nutrition education identified smartphones as the primary channel to access the Internet and health-related information among study participants [26]. The study emphasized the feasibility of developing an online nutrition education program for AI/AN adults [26].

While shortcomings in broadband infrastructure and high costs persist as barriers; AI/AN communities have reported high rates of mobile broadband use [27]. Recent studies have noted that recruitment of AI/AN communities using social media might expand the number of study participants due to the convenience offered by online platforms, which can alleviate the burden of finding transportation to attend sessions [28]. This was achieved in the evaluation of the BRAVE help-seeking intervention, which was delivered via text message (SMS) [29]. A total of 2330 AI/AN teens and young adults nationwide were successfully recruited using social media channels and text message posts. Out of the 1030 enrolled participants, 87% completed both intervention arms [29]. Such convenience is of crucial importance for parent-adolescent based interventions delivered in AI/AN settings [28].

### Overview of Talking Is Power Text Message Service

An interdisciplinary team who developed the Healthy Native Youth website, an online resource for practitioners who serve Native youth [30], developed the Talking is Power text-messaging service to guide parents and caring adults on how to initiate sensitive topics with youth and how to support them when making informed decisions regarding sex and healthy relationships [31]. Talking is Power is one of the first parental text-messaging sexual health interventions designed to improve parent–child communication among Native families [31]. The service was produced in collaboration by the Northwest Portland Area Indian Health Board, the Alaska Native Tribal Health Consortium, the Inter Tribal Council of Arizona, Inc., and the University of Texas Health Science Center at Houston. Parents and caregivers enroll for the service by texting EMPOWER to 97779 to receive up to three text messages per week with conversation starters, tips, video demonstrations, and words of encouragement. Topics covered in the text-messaging series include sexual health, pregnancy, STIs, and consent [31]. A complementary web-based resource page is also available as part of the Talking is Power initiative to provide parents with informational resources, including flyers, YouTube videos, and fact sheets addressing topics such as setting limits with your teen, creating boundaries, and goal setting [32]. These resources are listed under four categories—“Welcome Messages”; “The Future”; “The Rules”; and “Share”—with the goal of offering parents and caring adults a step-by-step process to communicate with their teens about sexual health and other sensitive topics [32].

This paper explores the following aims (1) to assess the use and reach of the Talking is Power web-based resource page using Google analytics; (2) to assess the usability of the Talking is Power text-messaging series based on users’ perspectives and engagement; (3) to evaluate the perceived impact of the Talking is Power text-messaging series in improving parental comfort to initiate sexual health conversations with youth; and (4) to highlight the main themes that emerged from parental feedback on the content and delivery of the text message series. Lessons learned during the preliminary design and evaluation of the SMS platform may be of interest to other communities who are interested in adapting or implementing similar virtual approaches.

## 2. Methods

### 2.1. Study Design and Population

We conducted a pilot study from 1 May 2020, to 29 April 2021, for the Talking is Power text-messaging series, which enrolled parents and caregivers of AI/AN youth across the United States, who were interested in acquiring skills to communicate with their teens about sexual health. A total of 375 parents signed up to receive the Talking is Power text-messaging series by 29 April 2021. Demographic information was collected for participants who completed the pre-survey (*n* = 99).

Participants were recruited via print materials and animated promotional videos, but recruitment was primarily conducted through advertisements placed on Healthy Native Youth’s social media channels due to the COVID-19 pandemic which was in full effect during the pilot study timeline to the present. The service was also promoted on Healthy Native Youth’s website as part of the resources and tools available to parents and health advocates. An example flyer is included in Figure S1. Recruitment posts included: “Join Talking is Power, a Text Messaging Service for parents and caring adults, that shares how to talk to youth about sexual health “Text “EMPOWER” to 97,779 to get started!” A paid press release service (Cision) was also used to expand the reach of the campaign leveraging print and online media outlets—Innovative Text Messaging Campaign Promotes Indigenous Youth Sexual Wellness—which was deployed in mid-February 2021. The service reports analytics 48 h after posting, including the number of news outlets that shared the press release, the number of press release views and hits, and the “potential audience” for each news outlet [33].

### 2.2. Data Collection and Management

To collect user demographics and preliminary feedback on the program, the developers included an optional, anonymous, pre- and post-survey. Without unique identifiers the pre- and post-surveys could not be matched at the individual level. The survey links were sent to participants via the “Talking is Power” text message service (MobileCommons), and were collected via SurveyMonkey, accessible only to the study primary investigator (PI-SCR). The pre-survey link was texted to participants after they signed up, and the post-survey link was texted to participants after they completed the series (eight weeks from start). A follow-up 15-day reminder was sent to all participants as a reminder to complete the survey if it had not been completed. There was no incentive for completing the pre- or post-survey. No names or tribal identifiers were collected. Out of 375 parents who signed up for the service (as of 29 April 2021), 99 parents completed the pre-survey, and 13 completed the post-survey. IP addresses were checked for quality assurance purposes and to avoid potential repeat respondents.

Data obtained by the PI (SCR) at the Northwest Portland Area Indian Health Board (NPAIHB) through the Healthy Native Youth website (Google Analytics) and pre- and post-surveys (SurveyMonkey) were de-identified prior to receipt and were secured in an encrypted folder that can only be accessed by the Co-PIs working on the project. The co-PI (LS) entered all coded data into the STATA database for analysis. All data management protections and protocols followed by the NPAIHB Tribal EpiCenter were used to protect participant data. Because program participants were adults and the survey questions involved no risk to participants, the study was approved as exempt research by the NPAIHB IRB. Participants were informed of the option to participate via text message, that included a link to the survey with an imbedded consent form. They could participate in the service whether or not they completed the feedback form(s). Participants were aware of the optional nature of the survey and that they could opt-out or leave the survey at anytime.

## 3. Measurements

Pre- and post-survey measures were developed based on similar pilot usability studies that do not require statistical significance to determine major usability problems [34,35,36,37,38,39,40]. Demographic information: The pre-survey collected demographic information from the respondent, including state of residence (“What state do you live in?”), gender (“What is your gender?”), and age of child/youth (“How old is your child/youth?”). State of residence was defined as a nominal categorical variable, including a list of 50 U.S. states. Gender was defined as a nominal categorical variable (male, female, transgender, other), while age of child/youth was defined as an ordinal categorical variable (elementary school, middle school, high school, older). Gender was coded as male = 0, female = 1, transgender = 2, and other = 3, while age of child/youth was coded as elementary school = 0, middle school = 1, high school = 2, and older = 3.

*Reach:* Reach of the Talking is Power text-messaging service was measured as the number of parents or caring adults who enrolled in the text-messaging series by month and during the period of data analysis. Google analytics were reviewed to assess page views for the Talking is Power page on the Healthy Native Youth website, average time spent on the page, entrances to the website, and bounce rates (web traffic analysis) between 1 May 2020 and 29 April 2021. The bounce rate was defined as the percentage of visitors who left the Talking is Power webpage without taking an action, such as clicking on a link or searching for resources on the series webpage [41]. While the exit rate is similar to the bounce rate, the difference between the two is that the former focuses on users who leave the Talking is Power page specifically rather than other webpages (i.e., Healthy Native Youth website) leading to the series page [41].

A separate analysis of reach was conducted for the press release by evaluating its total pick up by print and online news outlets, the press release’s views and hits, and the potential audience for each news site. Total pick up was measured as the number of exact matches and tweets found for Talking is Power [42]. Potential audience refers to the potential number of viewers exposed to the Talking is Power website and related tweets. It is calculated by the PR distribution service, as the sum of the average number of visitors (obtained from an audience data provider) who visited online sites promoting the Talking is Power series and the number of followers for each Twitter handle that tweets or retweets the Talking is Power release (obtained from Twitter) [42]. Release views and hits referred to the total number of views collected by the PR based on an aggregated estimate of the traffic to the Talking is Power website, including hits to the release on the PR newswire site from search engine crawlers [42]. Finally, engagement actions were measured as the total number of click-throughs, shares, and downloads related to the Talking is Power press release [42].

*Measures of Series Usability:* Five questions included in the post-survey aided in the assessment of the usability of the text-messaging series (Table 1) Usability was defined as whether the program was successful in attaining user satisfaction, whether the images, texts, links, and contents were engaging to users, whether the program related to users’ experience talking to youth, and whether the series helped spark sensitive conversations with youth. Participants were asked “How likely is it that you would recommend this series to a friend or colleague?” with response options ranging from 0 to 10. This question was coded as a continuous variable on a scale from 0 to 10, where 0 is the reference category. In addition, questions were asked in relation to how well the prompts (orientation to the series, background on the topics covered, videos watched with teens, images with reinforcing info, discussion prompts, encouraging messages and tips to talk about a sensitive topic) within the text message series resonated with participants (5-point Likert scale; extremely well–not at all well), the quality of the images, texts, and links (5-point Likert scale; very high quality–very low quality), and the likelihood of signing up for a similar series on a different topic (5-point Likert scale; extremely likely–not at all likely). A final question was asked regarding the series-related change in sparking sensitive conversations with youth/children and was coded as a categorical variable (no/yes/other), with the ability of participants to further specify what other changes were noted following completion of the series.

*Measure of Perceived Impact:* Perceived impact was measured as the change in parental comfort in initiating sexual health conversations after receiving the text message series using one item included in the pre- and post-survey.

Both surveys asked, “How comfortable are you talking with youth about sexual health topics?” with response options ranging from very to not at all on a 5-point Likert scale. The not at all category was used as the reference category and was coded as 0. This measure helped evaluate the impact of the series on the overall self-confidence of parents in initiating sexual health conversations with their children, by assessing changes in self-reported comfort. Changes in mean scores were calculated to assess whether any difference was observed.

*Participant Feedback:* Participants were given the opportunity to provide detailed feedback in the post-survey through an open-ended question (“We appreciate your honest feedback. If you could change or improve something about the series-what would it be?”) asking about their honest opinions, including suggestions on what should be modified or included in future versions. A similar open-ended question (“Do you have any other comments, questions, or concerns?”) was asked in the pre-survey to assess any comments, questions, or concerns that the participants might have regarding the platform in general or their participation in the Talking is Power series. Emerging themes were highlighted to guide future improvements.

## 4. Data Analysis

Descriptive statistics were used to demonstrate website analytics and participants’ responses to the pre- and post-survey questions. To assess the perceived impact of the series, the differences in mean percentage scores of the question assessing parental comfort in engaging in sexual health topics with youth between pre- and post-intervention were calculated using two-sample *t*-tests of equal variances. All statistical analyses were conducted using STATAIC 16. To categorize and highlight emerging themes from the open-ended feedback items, descriptive content analysis was conducted.

## 5. Results

### 5.1. Website and Text-Message Analytics and Service Reach

#### Talking Is Power Text-Message Service

From 1 May 2020 to 29 April 2021, the Talking is Power webpage received 1878 page views, with 862 unique visits (5.8% of HNY website’s total traffic). The average time spent on the page was 2 min and 23 s. The bounce rate was 73.1% (22.6% greater than the industry average), and the exit rate was 54.3% (15.2% greater than the industry average).

The paid press release that was deployed in mid-February 2021 was picked up (i.e., re-posted) by 94 Associated Press news outlets by mid-April, with a potential audience of 73 million. The press release online views and hits totaled 3326. The top five media outlets were MarketWatch (36 million visitors/month), Seeking Alpha (10 million visitors/month), Cision (8 million visitors/month), Benzinga (8 million visitors/month), and Morning Star (3 million visitors/month). While broad in reach, these news outlets are not well targeted to intended AI/AN audience.

## 6. Demographic Information

Table 2 describes the demographics of the participants who completed the pre-survey prior to the Talking is Power series. Ninety-nine participants completed the pre-survey. Of these, 89% (*n* = 87) were females, 10% were males (*n* = 10), and 1% (*n* = 1) identified as other (non-binary). Age of child/youth (*n* = 96) ranged between elementary school (19%, *n* = 18), middle school (30%, *n* = 29), high school (43%, *n* = 41), and older (8%, *n* = 8). Participants (*n* = 98) resided in 21 out of the 50 states in the U.S.

## 7. Talking Is Power Series Satisfaction and Usability

An adequate sample size of 13 participants completed the post-survey assessing series usability [43]. Five questions were included in the post-survey to assess satisfaction with the series (Table 1). When asked about the likelihood of recommending the series to a friend or colleague on a scale of 0–10 (*n* = 12), 17% (*n* = 2) of participants were classified as detractors (0–6), 8% (*n* = 1) of participants were classified as passives (7–8), and 50% (*n* = 6) of participants were classified as promoters (9–10).

Additionally, 85% (*n* = 11) of participants (*n* = 13) ranked highly (extremely likely *n* = 7; very likely *n* = 4) on the 5-point Likert scale regarding the likelihood of signing up for a similar series on a different topic. When asked about how well the prompts within the text message series related with them (*n* = 13) on a 5-point Likert scale, 70% (*n* = 9) ranked on the higher-end of the scale (extremely well *n* = 4; very well *n* = 5). The quality of the images (*n* = 13) ranked highly on the 5-Likert scale, with 62% (*n* = 8) of post-survey participants rating the quality of images, texts, and links as “very high quality”. Finally, 46% (*n* = 6) reported the text message series helped spark sensitive conversations with youth, 39% (*n* = 5) answered “other” and provided an open-ended response, and 15% (*n* = 2) answered “no.” The responses provided as “other” included parents being more comfortable to discuss sensitive topics with their children, youth feeling more comfortable to discuss sexual health topics with their parents, and youth asking their parents questions once they learned that their parents completed the series (Table 3).

## 8. Measure of Perceived Impact

A two-sample *t*-test with equal variances was carried out as a preliminary assessment of the impact of Talking is Power to check whether the mean of the difference in parental comfort levels changed between pre-and post-surveys. No significant difference in parental comfort levels was reported (*p* = 0.78 > 0.05). However, only 13 participants completed the post-survey out of the 99 who completed the pre-survey, which may have affected the results calculated.

## 9. Participant Feedback

Out of the 99 participants who completed the pre-survey, 26 provided open-ended responses on the program. Most respondents reported interest in the series and perceived it as helpful initiating conversations with youth. Some participants revealed interest to participate in similar series addressing other adolescent health topics. Another theme that emerged revolved around parental concerns about initiating sexual health conversations with youth and their children’s sexual activity, specifically during the COVID-19 pandemic (Table 4). When filling out the post-survey, seven out of 13 participants provided suggestions for improving the series, while others included positive feedback about the material, with the possibility of tailoring the series to other adolescent health topics (Table 5).

## 10. Discussion

This study is the first to assess the reach, usability, and perceived impact of a parental sexual health text-messaging service and web-based resource aiming to empower sensitive conversations between AI/AN parents and their teens. The development of the series and its complementary website were strengthened by involving diverse stakeholders in the design process, including tribal representatives, adolescent health educators, researchers, and federal agencies. Findings from this study highlight the preliminary positive impact of the program in fostering parental warmth, which has been attributed to youth well-being in the literature [44]. Such effective parent–child communication strategies could help increase adolescent disclosures about their sexual activity when discussing sexual health topics with their parents.

In response to COVID-19, the team had to pivot from in-person recruitment plans to virtual strategies. Traditional marketing activities at AI/AN conferences, trainings, and special events with the distribution of print materials were put on hold during the pandemic, hampering outreach. Solely relying on e-marketing via social media and paid advertising does not have the same traction or engagement amongst adults than in-person, traditional recruitment. Parents and caring adults prefer in-person community engagement as reported by previous programs [6,28]. The gap in audience reach was further highlighted by the paid press release. Only 94 news agencies circulated the article. Better targeting of the article to news outlets geared towards parents, educators, and sexual health interest groups could improve future recruitment efforts.

Despite these limitations, user enrollment and use of the online resources was strong. The average time spent on the Talking is Power page was 2 min and 23 s, 15% greater than the industry average (2 min and 4 s). The webpage links and attachments received 1878 pageviews, with 862 unique visits (5.8% of HNY website’s total traffic). However, the pages bounce rate was 73.1% (22.6% greater than the industry average), and the exit rate was 54.3% (15.2% greater than the industry average), leaving room for improvement. Industry averages are calculated via Google Analytics compared to similar websites [45]. Since only 5% of the site’s total traffic is currently attributed to Talking is Power, additional refinements could increase its visibility on the site, like adding Talking is Power links or widgets to the homepage.

Early feedback on the series was overwhelmingly positive, as 85% expressed interest in signing up for a similar series. Additionally, 70% of participants felt they could relate to the series’ prompts, and 62% ranked the quality of the images, links, and texts as “very high quality.” This finding emphasizes the importance of involving and engaging stakeholders in content development for improved program outcomes. Furthermore, around 61% (“yes” and “other”) reported that the text message prompts helped spark sensitive conversations with youth.

When assessing the perceived impact of the series on parental comfort initiating such conversations, there was no significant change in mean scores between the pre- and post-survey. This finding could be attributed to the limited number of participants who completed the post-survey (*n* = 13). While the study was underpowered to examine statistical differences, the sample size was consistent with previous usability testing protocols that do not require statistical significance to determine major usability problems [34,35,36,37,38,39,40]. Pilot studies are necessarily limited and aim to provide a measure of confidence that broader field trials are warranted and to highlight “red flags” (ranging from program bugs to deleterious usability or psychosocial impact) that suggest field testing is premature. The COVID-19 pandemic was also predicted to affect post-survey completion rates after observing a smaller completion rate than expected for the series. The reported results are an important first step to demonstrate the relative efficacy of this innovative text message series, as well as to determine areas of improvement before broader dissemination. Power analysis assessing relationships across variables was missing due to the limited number of post-hoc observations [46]. A larger sample size will be needed for future studies to evaluate the true impact of the series on parent–child communication.

Qualitative feedback collected in the pre-survey identified high parental interest in the Talking is Power series, which shows promise for the series uptake among AI/AN parents and caregivers. There may have been a tendency for parents and caregivers who are already comfortable initiating sexual health conversations with youth to sign up for Talking is Power. This calls for rigorous marketing efforts to reach a broader network of AI/AN caregivers. The post-survey feedback will guide future updates and improvements, which may in turn improve user engagement, website visits, and survey completion rates.

## 11. Limitations

This study had several limitations. Only one item was included in the pre- and post-survey to assess perceived impact of the series in increasing parental comfort to initiate sexual health conversations with their teens. This item can thus provide us with only a general overview of whether the series was able to initiate an impact on parental comfort after participation in the intervention. Another limitation was that the participants’ pre and post-intervention surveys were not matched, which can hinder the ability to conduct a repeated measures analysis and control for between-subject variability. Yet, assessing the difference in mean parental comfort level scores for the scope of this pilot study still provides important information for the overall impact of the text-messaging series in initiating sensitive sexual health conversations with youth. Follow-up studies can use demographic information as an identifier for participants who completed both the pre- and post-surveys.

Both the pre- and post-surveys and usability tests involved relatively small sample sizes that may not be representative of all AI/AN parents in the United States who have participated in the intervention. However, ongoing enrollment of parents, public relations services, and marketing efforts are expected to increase reach and can contribute to the further assessment of the series and its website in the future. Finally, this study focuses on descriptive statistics as compared to in-depth statistical testing due to the preliminary stages of the intervention.

## 12. Conclusions

Given existing disparities in adolescent sexual health in AI/AN communities and the protective role of parents and caregivers against early sexual debut, the Talking is Power text message series holds great promise as a means to empower parents to initiate sensitive sexual health topics with their youth and promote informed decision-making. Lessons learned during the design, dissemination, and evaluation of the Talking is Power resource may be of interest to other communities interested in adapting or implementing similar approaches.

## Figures and Tables

**Table 1:** Questions for Post-Survey Satisfaction and Usability.

Series Usability Question	Scale
How likely is it that you would recommend this series to a friend or colleague?	0–10
How well do the prompts relate to you?	5-point Likert scale (Extremely well–Not at all well)
How would you rate the quality of the images, text, and links?	5-point Likert scale (Very high quality–Very low quality)
How likely are you to sign up for a similar series on a different topic?	5-point Likert scale (Extremely likely–Not at all likely)
Did the series help spark sensitive conversations with your child/young adult?	(No change/Yes, we’re talking more/Other changes)

**Table 2:** Demographic Characteristics of Talking is Power Participants.

Demographic Variables	Frequency (*n*)	*n* (%)	Mean (SD)
Gender (*n* = 98) *			0.9184 (0.3709)
Male	10	10	
Female	87	89	
Other	1	1	
Age of Child/Youth (*n* = 96) *			1.4063 (0.8894)
Elementary School	18	19	
Middle School	29	30	
High School	41	43	
Older	8	8	
State of Residence (*n* = 98) *			
Alaska	15	16	
Washington	13	13	
Oregon	13	13	
California	12	12	
Oklahoma	11	11	
New Mexico	9	10	
Michigan	3	3	
Arizona	3	3	
Montana	3	3	
Florida	3	3	
Wyoming	2	2	
Wisconsin	2	2	
Arkansas	1	1	
North Carolina	1	1	
Iowa	1	1	
Missouri	1	1	
Nebraska	1	1	
Virginia	1	1	
New York	1	1	
Texas	1	1	
Minnesota	1	1	

* Note that some values were missing for the total number of participants (*n* = 99).

**Table 3:** Measures for Post-Survey Series Usability.

Series Usability Category	Scale	Post-Test (*n*%)
Recommend series to a friend (*n* = 12)	0–10	0–6 (17%)
		7–8 (8%)
		9–10 (75%)
Relation to prompts (*n* = 13)	5-point Likert scale (*Extremely well-Not at all well*)	Not at all well 0 (0%)
		Not so well 2 (15%)
		Somewhat well 2 (15%)
		Very well 5 (39%)
		Extremely well 4 (31%)
Quality of Images Texts and Links (*n* = 13)	5-point Likert scale (*Very high quality-Very low quality*)	Very low quality 0 (0%)
		Low quality 0 (0%)
		Neither high nor low quality 2 (15%)
		High quality 3 (23%)
		Very high quality 8 (62%)
Sign up for similar series (*n* = 13)	5-point Likert scale (*Extremely likely-Not at all likely*)	Not at all likely 1 (8%)
		Not so likely 0 (0%)
		Somewhat likely 1 (8%)
		Very likely 4 (30%)
		Extremely likely 7 (54%)
Spark sensitive conversations (*n* = 13)	(No change/Yes, we’re talking more/Other changes)	No change 2 (15%)
		Yes, we’re talking more 6 (46%)
		Other changes * 5 (39%)

* Changes included parents being more comfortable to discuss sensitive topics with their children, youth feeling more comfortable to discuss sexual health topics with their parents, and youth asking their parents questions once they learned that their parents completed the series.

**Table 4:** Pre-survey Participant Feedback Qualitative Themes and Quotes.

Pre-survey Participant Feedback	Major Themes	Quotations
	Series helpful in initiating sensitive conversations with youth	*“I manage a local health department’s sexual health program-seeking to learn more and better serve our whole community.”* *“Always looking for ways to educate my sons on sexual health, relationships, etc.”* *“I’m a therapist, and sometimes topics come up. This is an excellent referral source. Thank you.”* *“We also work with native youth K-12 so this will be helpful.”* *“Thank you for this service! I think it will be a good reminder to keep these important conversations going with our children.”*
	Potential topics that could be addressed in similar series	“*Peer pressure and bullying.”* *“Topics about discovering themselves and healing for youth that have went through traumatic events”.* *“Suicide/depression. Both my sons isolate themselves. I have a 16 and 21-year-old that is still in the home. Have set them up with therapists during COVID-19 but doesn’t seem to be helpful. I’m a single mother and work long, exhausting days. Very worried about their well-being and want to help them with being socially and physically well.”* *“Talking about what to do with an unwanted teen pregnancy and abortion is hard. I am prochoice.”* *“My teen has reactive attachment disorder. She has self-harmed once. She has said she prefers to be sad and angry by her own report. We need ways to encourage positive thinking and more genuine emotions other than sadness and anger.”* *“Impact of social media presence on our students.”*
	Concerns about sexual health conversations and youth sexual activity	*“What age do we start talking to youth about the MMIW movement? I want youth aware, but I also don’t want to cause fear or trauma in speaking about it. Thanks!”* *“I don’t know how to begin the conversation with my eldest niece who is 17 going on 18.”* *“My son has a developmental delay, and I get concerned things such as his sexuality of sexual activity will get warped or lost for him.”* *“Concerned about social choices during the COVID19 lockdown restrictions lifting.”*

**Table 5:** Post-survey Participant Feedback Qualitative Themes and Quotes.

Post-survey Participant Feedback	Major Themes	Quotes
	Improving mode of series delivery	*“Get a live person to talk with people doing the course as they do it. Avatar X never talked to me.”* *“Longer time in between messages? Longer overall. Thank you for the support!”* *“Send out more alerts.”* *“One thing that I have yet to come across is how to talk to a child who has intellectual or other disabilities. I modify as much as I can, but it would be helpful if you guys somehow incorporated that into this lesson. Thank you I appreciated this-helped me out and kept me current with today’s slang topics etc.”*
	Positive feedback/Other adolescent health topics	*“Nothing. I like the frequency, the reminders and all the materials. Thank you!”* *“It would be nice to have this series address other adolescent health topics.”*

## Data Availability

The data presented in this study are available on request from the corresponding authors. The data are not publicly available for participant privacy purposes.

## Supplementary Materials

The following are available online at https://www.mdpi.com/article/10.3390/ijerph18179126/s1, Figure S1: A Sample of *Talking is Power* Promotional Materials (Flyer).

## References

[1] Martin J.A., Hamilton B.E., Osterman M.J., Curtin S.C., Matthews T.J. (2015). Births: Final data for 2013. National vital statistics reports: From the Centers for Disease Control and Prevention, National Center for Health Statistics. Natl. Vital Stat. Syst..

[2] Centers for Disease Control and Prevention (2017). STDs in Racial and Ethnic Minorities. Division of STD Prevention, National Center for HIV/AIDS, Viral Hepatitis, STD, and TB Prevention.

[3] Centers for Disease Control and Prevention and Indian Health Service (2012). Indian Health Surveillance Report—Sexually Transmitted Diseases 2009.

[4] De Ravello L., Tulloch S., Taylor M. (2012). We will be known forever by the tracks we leave: Rising up to meet the reproductive health needs of American Indian and Alaska Native youth. Am. Indian Alsk. Nativ. Ment. Health Res..

[5] Kaufman C.E., Shelby L., Mosure D.J., Marrazzo J., Wong D., de Ravello L., Rushing S.C., Warren-Mears V., Neel L., Eagle S.J. (2007). Within the Hidden Epidemic: Sexually Transmitted Diseases and HIV/AIDS Among American Indians and Alaska Natives. Sex. Transm. Dis..

[6] Craig Rushing S., Gaston A., Kaufman C., Markham C., Jessen C., Gorman G., Torres J., Black K., Shegog R., Koogei Revels T., Dyson L.E., Grant S., Hendriks M. (2016). Using technology to promote health and wellbeing among American Indian and Alaska native teens and young adults. Indigenous People and Mobile Technologies.

[7] Fuentes M., Garcia O., Garcia F. (2020). Protective and Risk Factors for Adolescent Substance Use in Spain: Self-Esteem and Other Indicators of Personal Well-Being and Ill-Being. Sustainability.

[8] Fuentes M.C., Alarcón A., García F., Gracia E. (2015). Use of alcohol, tobacco, cannabis and other drugs in adolescence: Effects of family and neighborhood. An. Psicol..

[9] Martinez I., Garcia F., Musitu G., Yubero S. (2012). Family socialization practices: Factor confirmation of the Portuguese version of a scale for their measurement. Rev. Psicodidact..

[10] Gallarin M., Torres-Gomez B., Alonso-Arbiol I. (2021). Aggressiveness in Adopted and Non-Adopted Teens: The Role of Parenting, Attachment Security, and Gender. Int. J. Environ. Res. Public Health.

[11] Ridao P., López-Verdugo I., Reina-Flores C. (2021). Parental Beliefs about Childhood and Adolescence from a Longitudinal Perspective. Int. J. Environ. Res. Public Health.

[12] Chen C.S., Farruggia S. (2002). Culture and Adolescent Development. Online Read. Psychol. Cult..

[13] Darling N., Steinberg L. (1993). Parenting style as context: An integrative model. Psychol. Bull..

[14] Branstetter S.A., Furman W. (2012). Buffering Effect of Parental Monitoring Knowledge and Parent-Adolescent Relationships on Consequences of Adolescent Substance Use. J. Child Fam. Stud..

[15] Whitesell N.R., Kaufman C.E., Keane E.M., Crow C.B., Shangreau C., Mitchell C.M. (2012). Patterns of substance use initiation among young adolescents in a Northern Plains American Indian tribe. Am. J. Drug Alcohol Abuse.

[16] Griese E.R., Kenyon D.B., McMahon T.R. (2016). Identifying sexual health protective factors among Northern Plains American Indian youth: An ecological approach utilizing multiple perspectives. Am. Indian Alsk. Nativ. Ment. Health Res..

[17] (2016). Power to Decide (Formerly the National Campaign to Prevent Teen and Unplanned Pregnancy). Survey Says: Parent Power.

[18] Chewning B., Douglas J., Kokotailo P.K., Lacourt J., Clair D.S., Wilson D. (2001). Protective factors associated with American Indian adolescents’ safer sexual patterns. Matern. Child Health J..

[19] Hellerstedt W.L., Peterson-Hickey M., Rhodes K.L., Garwick A. (2006). Environmental, Social, and Personal Correlates of Having Ever Had Sexual Intercourse Among American Indian Youths. Am. J. Public Health.

[20] Stanton B., Cole M., Galbraith J., Li X., Pendleton S., Cottrell L., Marshall S., Wu Y., Kaljee L. (2004). Randomized trial of a parent intervention: Parents can make a difference in long-term adolescent risk behaviors, perceptions, and knowledge. Arch. Pediatric Adolesc. Med..

[21] Wu Y., Stanton B.F., Galbraith J., Kaljee L., Cottrell L., Li X., Harris C.V., D’Alessandri D., Burns J.M. (2003). Sustaining and Broadening Intervention Impact: A Longitudinal Randomized Trial of 3 Adolescent Risk Reduction Approaches. Pediatrics.

[22] Santa Maria D., Markham C., Bluethmann S., Mullen P.D. (2015). Parent-based adolescent sexual health interventions and effect on communication outcomes: A systematic review and meta-analyses. Perspect. Sex. Reprod. Health.

[23] Sequist T.D., Cullen T., Acton K.J. (2011). Indian Health Service Innovations Have Helped Reduce Health Disparities Affecting American Indian And Alaska Native People. Health Aff..

[24] Carroll M., Cullen T., Ferguson S., Hogge N., Horton M., Kokesh J. (2011). Innovation in Indian Healthcare: Using Health Information Technology to Achieve Health Equity for American Indian and Alaska Native Populations. Perspect. Health Inf. Manag..

[25] Hiratsuka V.Y., Moore L., Avey J.P., Dirks L.G., Beach B.D., Dillard D.A., Novins D.K., Pearson C., El-Khatib Z. (2019). An Internet-Based Therapeutic Tool for American Indian/Alaska Native Adults with Posttraumatic Stress Disorder: User Testing and Developmental Feasibility Study. JMIR Form. Res..

[26] Stotz S., Brega A.G., Lockhart S., Hebert L.E., Henderson J.N., Roubideaux Y., Moore K. (2021). An online diabetes nutrition education programme for American Indian and Alaska Native adults with type 2 diabetes: Perspectives from key stakeholders. Public Health Nutr..

[27] National Telecommunication and Information Administration (2014). Exploring the Digital Nation: Embracing the Mobile Internet. https://www.ntia.doc.gov/files/ntia/publications/exploring_the_digital_nation_embracing_the_mobile_internet_10162014.pdf.

[28] Kaufman C.E., Schwinn T.M., Black K., Keane E.M., Crow C.K.B. (2016). The Promise of Technology to Advance Rigorous Evaluation of Adolescent Pregnancy Prevention Programs in American Indian and Alaska Native Tribal Communities. Am. J. Public Health.

[29] Stephens D., Peterson R., Singer M., Johnson J., Rushing S.C., Kelley A. (2020). Recruiting and Engaging American Indian and Alaska Native Teens and Young Adults in a SMS Help-Seeking Intervention: Lessons Learned from the BRAVE Study. Int. J. Environ. Res. Public Health.

[30] Craig Rushing S., Stephens D., Shegog R., Torres J., Gorman G., Jessen C., Gaston A., Williamson J., Tingey L., Lee C. (2018). Healthy Native Youth: Improving Access to Effective, Culturally-Relevant Sexual Health Curricula. Front. Public Health.

[31] Healthy Native Youth (2020). Talking is Power: A Text Messaging Service for Parents and Caring Adults. https://www.healthynativeyouth.org/wp-content/uploads/2020/03/One-Pager_TalkingIsPower_FINAL.pdf.

[32] Healthy Native Youth (2020). Talking is Power: Tools for Parents. https://www.healthynativeyouth.org/resources/talking-is-power-tools-for-parents/.

[33] 13 Important Metrics for Your Press Release Distribution. https://skograndpr.com/2018/09/20/13-important-metrics-for-your-press-release-distribution/.

[34] Escobar-Chaves S.L., Shegog R., Moscoso-Alvarez M.R., Markham C., Tortolero-Luna G., Peskin M., Tortolero S. (2011). Cultural Tailoring and Feasibility Assessment of a Sexual Health Middle School Curriculum: A Pilot Test in Puerto Rico. J. Sch. Health.

[35] Faulkner L. (2003). Beyond the five-user assumption: Benefits of increased sample sizes in usability testing. Behav. Res. Methods Instruments Comput..

[36] Markham C.M., Shegog R., Leonard A.D., Bui T.C., Paul M.E. (2009). +CLICK: Harnessing web-based training to reduce secondary transmission among HIV-positive youth. AIDS Care.

[37] Nielsen J. (1995). Usability Engineering.

[38] Shegog R., Peskin M.F., Markham C., Thiel M., Karny E., Addy R.C., Johnson K.A., Tortolero S. (2014). It’s Your Game-Tech: Toward Sexual Health in the Digital Age. Creat. Educ..

[39] Shegog R., Markham C.M., Leonard A., Bui T., Paul M. (2011). “+CLICK”: Pilot of a web-based training program to enhance ART adherence among HIV-positive youth. AIDS Care.

[40] Shegog R., Markham C.M., Peskin M., Dancel M., Coton C., Tortolero S. (2007). “It’s Your Game”: An innovative multimedia virtual world to prevent HIV/STI and pregnancy in middle school youth. Stud. Health Technol. Inform..

[41] What Is Bounce Rate? And How to Quickly Improve It. https://backlinko.com/hub/seo/bounce-rate.

[42] Visibility Reports—Metric Definitions. http://vrhelp.mediaroom.com/definitions.

[43] Turner C.W., Lewis J.R., Nielsen J., Karwowski W. (2006). Determining Usability Test Sample Size. International Encyclopedia of Ergonomics and Human Factors.

[44] Gimenez-Serrano S., Garcia F., Garcia O.F. (2021). Parenting styles and its relations with personal and social adjustment beyond adolescence: Is the current evidence enough?. Eur. J. Dev. Psychol..

[45] RankMonsters (2019). Website Analytics Benchmarks. https://rankmonsters.org/website-analytics-benchmarks/.

[46] García J.F., Pascual J., Frías M.D., Van Krunckelsven D., Murgui S. (2008). Design and power analysis: n and confidence intervals of means. Psicothema.

