# Supplementary material for: Assessment of the Reach, Usability, and Perceived Impact of “Talking Is Power”: A Parental Sexual Health Text-Messaging Service and Web-Based Resource to Empower Sensitive Conversations with American Indian and Alaska Native Teens
Source: Int J Environ Res Public Health. 2021 Aug 30;18(17):9126. doi: 10.3390/ijerph18179126 (PMC8431363; doi:10.3390/ijerph18179126)

Figure S1. Example of Recruitment Flyer for *Talking is Power*

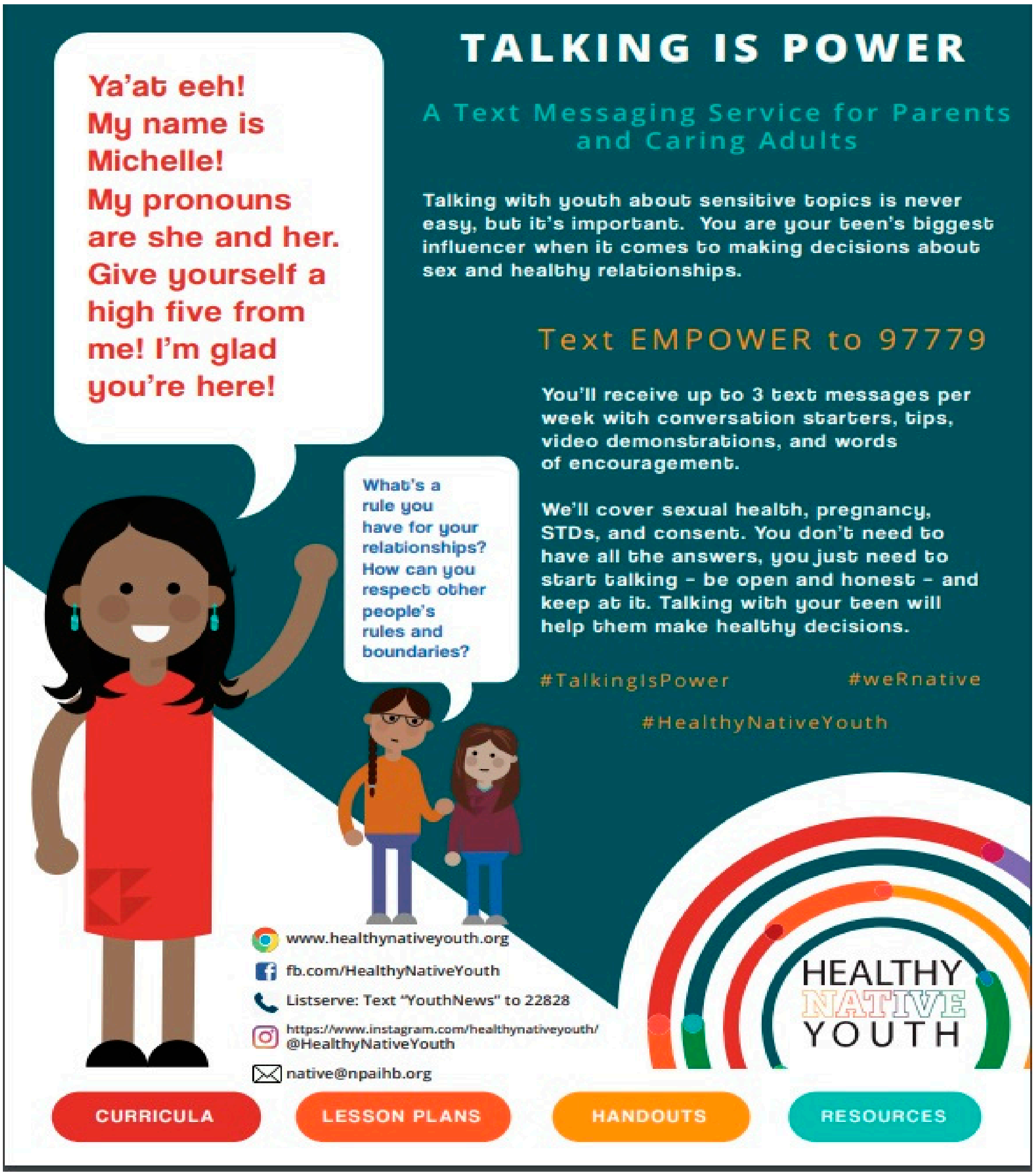

Supplement: Supplementary file 1 [file ijerph-18-09126-s001.zip › ijerph-1289883-supplementary.pdf]
